# The immune receptors TLR4 and SLAMF1 regulate TNF release by human metapneumovirus in human macrophages

**DOI:** 10.3389/fimmu.2025.1697494

**Published:** 2025-11-19

**Authors:** Katja Sæterhaug Bye, Kristin Rian, Liv Ryan, Terje Espevik, Marit W. Anthonsen, Maria Yurchenko

**Affiliations:** 1 Department of Clinical and Molecular Medicine, Norwegian University of Science and Technology, Trondheim, Norway; 2 Department of Infectious Diseases, Clinic of Medicine, St. Olavs Hospital HF, Trondheim University Hospital, Trondheim, Norway

**Keywords:** virus, airway disease, hyperinflammation, TNF, toll-like receptor 4, HMPV, SLAMF1

## Abstract

**Background:**

Human metapneumovirus (HMPV) is a major cause of acute respiratory disease in children, the elderly, and immunocompromised individuals. While pro-inflammatory cytokines and type I interferons (IFNs) are important for antiviral defense, excessive tumor necrosis factor (TNF) is associated with severe disease in HMPV and other respiratory infections. Hence, defining regulatory mechanisms by which HMPV induces TNF and IFN-β is important for therapeutic strategies in airway disease. The immunoregulatory receptors Toll-like receptor (TLR)4 and signaling lymphocytic activation molecule family 1 (SLAMF1) mediate TNF and IFN-β expression in response to LPS and Gram-negative bacteria, but their involvement in HMPV-stimulated cytokine expression is unclear.

**Methods:**

We investigated the kinetics of *TNF* and *IFNB1* expression in human monocyte-derived macrophages (MDMs) and THP-1 macrophage-like cells. The impact of SLAMF1 and TLR4 on *TNF*, *IFNB1*, and p38 MAPK was determined after their overexpression or knockout in THP-1 cells or silencing in MDMs.

**Results:**

TLR4 knockout reduced *TNF* but not *IFNB1* induced by HMPV, whereas SLAMF1 silencing reduced both cytokines. Overexpression of TLR4 or SLAMF1 enhanced p38 MAPK activation and TNF secretion, while silencing of TLR4 or SLAMF1 reduced p38 MAPK activation and TNF secretion. Pharmacological inhibition of p38 MAPK reduced both *TNF* and *IFNB1*, confirming its essential role in cytokine induction.

**Conclusions:**

Together, our findings identify TLR4 and SLAMF1 as key regulators of early HMPV-induced inflammation via p38 MAPK. SLAMF1 additionally influences IFN-β responses and appears to affect viral replication dynamics. These insights suggest that targeting SLAMF1–TLR4 signaling may offer a therapeutic strategy to limit TNF-driven pathology in HMPV infection.

## 1 Introduction

Human metapneumovirus (HMPV) is an important human pathogen that can cause severe lower respiratory tract disease in infants, the elderly, and immunocompromised individuals, manifesting as bronchiolitis, pneumonia, or exacerbations of asthma or chronic obstructive pulmonary disease (1–3). HMPV is a negative−sense, single−stranded RNA virus of the *Pneumoviridae* family, with respiratory syncytial virus (RSV) being its closest relative (4). Reinfections with HMPV occur commonly throughout life (5), yet no vaccine or specific treatment for HMPV infection is currently available.

The innate immune system in the lungs detects viral infections through pattern recognition receptors (PRRs) expressed on cells such as airway epithelial cells and alveolar macrophages. These PRRs sense pathogen−associated molecular patterns (PAMPs) on viruses—such as surface glycoproteins, genomes, and replication intermediates—triggering an immediate immune response involving interferons (IFNs) and other inflammatory mediators that limit viral spread and recruit additional immune cells (6, 7). Depending on the infected cell type, different PRRs may be activated by HMPV to induce innate immune responses: the RIG−I−like receptors (RLRs); the endosomal Toll−like receptors (TLRs) TLR3, TLR7, and TLR8 (8, 9); and the LPS/Gram−negative bacteria sensing TLR4 (10, 11). Specific PRRs can generate distinct cytokine profiles depending on the downstream signaling complexes they engage. For instance, TLR4 can signal through either TIRAP/MyD88 or TRAM/TRIF complexes, leading to the production of pro−inflammatory cytokines or type I IFNs, respectively. TLR3 activation by its ligand preferentially promotes type I IFN production (12).

While respiratory epithelial cells are the primary targets of HMPV infection (13), HMPV can also infect dendritic cells and macrophages in the lungs (9, 14–16). Along with the main type I IFN-producing respiratory epithelial cells, alveolar macrophages initiate immune responses to HMPV by inducing type I IFNs and other pro−inflammatory cytokines, which generally contribute to viral clearance (17, 18). Macrophages are central regulators of immune balance and disease severity during infections with HMPV, RSV, influenza, and severe acute respiratory syndrome coronavirus 2 (SARS-CoV-2) (14, 18–21).

Macrophages are well known as potent producers of TNF, particularly in inflammatory conditions and in response to PAMPs (22). TLR4 is crucial for TNF induction by bacteria and by LPS, a component of Gram−negative bacteria (12). Importantly, although TNF is a key pro−inflammatory cytokine critical for antiviral defenses, it also mediates hyperinflammation and acute lung injury in respiratory viral infections, as reported for influenza virus and SARS−CoV−2 (23–27). Similarly, we and others have shown that HMPV infection is accompanied by elevated TNF levels in nasopharyngeal aspirates and blood serum, which are associated with greater disease severity in hospitalized children (24, 28). However, the regulatory mechanisms underlying HMPV−induced TNF production remain incompletely understood.

A growing list of viruses is recognized to induce inflammatory responses via TLR4, including RSV, SARS−CoV−2, Ebola virus, influenza virus, and HMPV (10, 11, 29–34). TLR4 can sense viral surface glycoproteins and initiate downstream signaling that drives pro−inflammatory cytokine production (10, 31, 35, 36). For RSV and SARS−CoV−2, excessive TLR4 signaling has been implicated in lung pathology and heightened disease severity (33, 37).

We recently identified that the immune cell receptor SLAMF1 (signaling lymphocytic activation molecule family 1) enhances TLR4−mediated TNF and IFN-β induction by *Escherichia coli* in human macrophages (38). SLAMF1 is a co−stimulatory molecule expressed by immune cells, including macrophages, and regulates signal transduction networks essential for effective immune responses (39–44). Whether SLAMF1 regulates HMPV−induced TNF and IFN-β expression has not been explored. In this study, we set out to characterize the contribution of TLR4 and SLAMF1 to the induction of TNF and IFN-β in HMPV−infected human primary macrophages. Our results demonstrate that both TLR4 and SLAMF1 regulate an early HMPV−stimulated upregulation of the p38 MAPK–TNF axis, while TLR4 does not affect IFN-β induction in human macrophages. These findings reveal novel mechanisms controlling TNF and IFN-β output in human macrophages, which may be relevant for therapeutic strategies targeting pro−inflammatory signaling in HMPV−infected patients.

## 2 Materials and methods

### 2.1 Cell culture and inhibitors

The use of human buffy coats and serum from the blood bank at St. Olavs Hospital (Trondheim, Norway) was approved by the Regional Committee for Medical and Health Research Ethics (REC) in Central Norway (#2009/2245). Primary human monocytes were isolated from the buffy coat by adherence, as previously described (45). In brief, freshly prepared buffy coats were diluted by 100 mL of PBS and applied on top of Lymphoprep (Axis-Shield, Dundee, Scotland) according to the manufacturer’s instructions. Cells were counted using Z2 Coulter particle count and size analyzer (Beckman Coulter, Munich, Germany) on program B, resuspended in RPMI 1640 (Sigma-Aldrich, Merck, Darmstadt, Germany) supplemented with 5% of pooled human serum at a concentration of 6 × 10^6^ per mL, and seeded to 24-well (0.5 mL per well) cell culture dishes. After a 60-min incubation for the surface adherence of monocytes, the dishes were washed three times with HBSS (Sigma-Aldrich, Merck) to remove non-adherent cells. Isolated cells were kept in RPMI 1640 medium supplemented with 10% human serum, 0.34 mM of L-glutamine, and 25 ng/mL of rhM-CSF (216-MC-025; R&D Systems, BioNordika, Oslo, Norway). THP-1 cells (ATCC) and generated THP-1 sublines were cultured in RMPI 1640 supplemented by 10% heat-inactivated FCS, 100 nM of penicillin/streptomycin (Thermo Fisher Scientific, Oslo, Norway), 5 μM of β-mercaptoethanol, 2 mM of L-glutamine, 10 mM of HEPES, 1 mM of sodium pyruvate, 4,500 mg/L of glucose, and 1,500 mg/L of sodium bicarbonate (Sigma-Aldrich, Merck). THP-1 cells (300,000 cells per/well, 24-well plates, in 0.5 mL of media per well) were differentiated with 60 ng/mL of PMA (Sigma-Aldrich, Merck) for 24 h, followed by 48 h in medium without PMA. The p38 MAPK inhibitor BIRB796 (doramapimod; Selleck Chemicals, #S1574, VWR International LLC, Norway) was dissolved in DMSO, aliquoted, and stored at −80°C to prevent repeated freeze–thaw cycles.

### 2.2 Generation of THP-1 sublines

For making TLR4 KO THP-1 and control THP-1 sublines, LentiCRISPRv2 plasmid (a gift from F. Zhang; 52961; Addgene; Sanjana et al., 2014) was ligated with 5′-AAACGCGTGAGACCAGAAAGCTGGC-3′ and 5′-CACCGAAGGTCCAAGTGCTCTAGAT-3′ for *TLR4* and 5′-CACCGTTTGTAATCGTCGATACCC3′ and 5′-AAACGGGTATCGACGATTACAAAC-3′ for control non-targeting guiding RNA expression. Generation of TLR4 overexpressing control cells is described in (46). For SLAMF1 overexpression, SLAMF1^Flag^ cDNA (38) was recloned to pLVX-EF1α-IRES-ZsGreen1 (Takara Bio, AH Diagnostics AS, Oslo, Norway). Packaging plasmids pMD2.G and psPAX2 were used for producing lentivirus (provided by D. Trono, École Polytechnique Fédérale de Lausanne, Lausanne, Switzerland; 12260 and 12259; Addgene). HEK293T cells (cultured in DMEM supplemented by 10% FCS, 100 IU/mL of penicillin, 100 μg/mL of streptomycin) were co-transfected with the packaging and lentiCRISPRv2 or the above-listed lentiviral pLVX constructs (empty vectors, or TLR4^Flag^ pLVX, or SLAMF1^Flag^ pLVX) using the GeneJuice transfection reagent (Merck), and the media was changed in 16 h. The lentivirus-containing supernatants were collected after another 48 h and used for transduction of THP-1 cells along with 8 µg/mL of protamine sulfate. Transduced THP-1 cells were then sorted based on ZsGreen co-expression 1 week after transduction using the BD FACS Aria III cell sorter (BD Biosciences, Oslo, Norway) with BD FACSDiva 8.0 software (BD Biosciences). All cell lines were regularly checked for mycoplasma contamination.

### 2.3 Virus propagation

Virus propagation was performed as described (16). Briefly, LLC-MK2 cells were inoculated with the clinical HMPV isolate NL/17/00 (A2) at a multiplicity of infection (MOI) of 0.01 in OptiMEM containing 2% FBS, 20 µg/mL of gentamicin, and 0.7 nM of glutamine. On days 7–8, the virus was harvested by freeze–thawing at −80°C, followed by purification on a 20% sucrose cushion and resuspension in OptiMEM (2% FBS). The virus titer was determined using a cell-based immunoassay. Purified virus particles were serially diluted (log10) on monolayers of LLC-MK2 cells in 96-well flat-bottom plates. After 4 days, cells were washed and stained with LIGHT DIAGNOSTICS™ HMPV direct fluorescence assay (Merck Millipore, Darmstadt, Germany), and foci-forming units were determined by manual counting.

### 2.4 *In vitro* HMPV infection

Cells were infected with HMPV A2 at MOI 1 in OptiMEM containing 2% FBS, 20 µg/mL of gentamicin, and 0.7 nM of glutamine. Cells were incubated with the virus for the indicated time.

### 2.5 siRNA treatment

Oligonucleotides used for silencing were AllStars negative control siRNA (SI03650318), FlexiTube siRNA Hs_SLAMF1_2 (SI00047250), and Hs_TLR4_2 (SI00151011; QIAGEN, Sollentuna, Sweden). On day 6 from isolation, cells were transfected with silencing oligonucleotides (20 nM final concentration) using Lipofectamine 3000 (L3000008, Invitrogen, Thermo Fisher Scientific, Oslo, Norway) as suggested by the manufacturer. Media was changed on day 7, transfection was repeated on day 8, media was changed on day 9, and HMPV infection was performed on day 10 to day 11.

### 2.6 qRT-PCR

Total RNA was isolated from the cells using QIAzol reagent (QIAGEN), and chloroform extraction was followed by purification on RNeasy Mini columns with DNAse digestion step (QIAGEN). cDNA was prepared with a Maxima First Strand cDNA Synthesis Kit (Thermo Fisher Scientific), in accordance with the protocol of the manufacturer, using 400 ng of total RNA per sample. qPCR was performed with the PerfeCTa qPCR FastMix (Quanta Biosciences, VWR International, Oslo, Norway) in replicates and cycled in a StepOnePlus Real-Time PCR cycler (Thermo Fisher Scientific). The following TaqMan Gene Expression Assays (Applied Biosystems, Thermo Fisher Scientific, Oslo, Norway) were used: *IFNβ* (Hs01077958_s1), *TNF* (Hs00174128_m1), *TBP* (Hs00427620_m1), *SLAMF1* (Hs00900288_m1), *TLR4* (Hs00152939_m1), and *CXCL10* (Hs01124251_g1). HMPV vRNA expression was analyzed by qRT-PCR using SybrGreen-based master mix Fast SYBR™ Green Master Mix (Thermo Fisher Scientific) and the following primers: HMPV-N (fwd) CATATAAGCATGCTATATTAAAAGAGTCTC, HMPV-N (rev) CCTATTTCTGCAGCATATTTGTAATCAG for *HMPV-N* and *TBP* (fwd) 5′-GAGCCAAGAGTGAAGAACAGTC-3′ and (rev) 5′-GCTCCCCACCATATTCTGAATCT-3′. Fold change in HMPV vRNA expression was calculated relative to the indicated infected sample in the figure legend. The level of *TBP* mRNA was used for normalization, and the results were presented as a relative expression compared with the control’s untreated sample. Relative expression was calculated using Pfaffl’s mathematical model (47). Graphs and statistical analyses were made with GraphPad Prism v9.1.2 (Dotmatics, Boston, MA, USA), with additional details provided in the figure legends and in Section 2.9.

### 2.7 Western blotting

Cell lysates for Western blotting (WB) analysis were prepared by simultaneous extraction of proteins and total RNA using QIAzol reagent (QIAGEN) as suggested by the manufacturer. The extracted total RNA was used for qRT-PCR, whereas protein samples were used for simultaneous analysis of protein expression/posttranslational modifications. Protein pellets after protein isolation were dissolved by heating the samples for 10 min at 95°C in a buffer containing 4 M of urea, 1% SDS (Sigma-Aldrich, Merck), and NuPAGE LDS Sample Buffer (4X) (Thermo Fisher Scientific), with a final 25 mM DTT in the samples. For SDS-PAGE, we used pre-cast gradient 4%–12% Bis-Tris protein gels NuPAGE Novex and 1X MOPS SDS running buffer (Thermo Fisher Scientific). Proteins from the gel were transferred to iBlot Transfer Stacks by using the iBlot Gel Transfer Device (Thermo Fisher Scientific). The blots were developed with the SuperSignal West Femto (Thermo Fisher Scientific) and visualized with the LI-COR ODYSSEY Fc Imaging System (LI-COR Biotechnology, Bad Homburg, Germany). For densitometry analysis of the WB bands, Odyssey Image Studio 5.2 software (LI-COR Biotechnology) was used, and the relative numbers of the bands’ intensity were normalized to the intensities of the respective loading-control protein (β-tubulin).

### 2.8 Antibodies and ELISA

The following primary antibodies were used for Western blotting: rabbit β-tubulin (ab6046) from Abcam; phospho-p38 MAPK (T180/Y182) (D3F9), phospho-STAT1 (Tyr701) (58D6), and anti-DYKDDDDK tag (D6W5B)/Flag tag from Cell Signaling Technology; mouse anti-HMPV Nucleoprotein from Abcam; and mouse STAT1 antibodies from BD Biosciences (#610185, Wokingham, UK). Secondary antibodies (HRP-linked) were from DAKO Denmark A/S (Glostrup, Denmark). The following ELISA assays were used: human CXCL10/IP-10 DuoSet ELISA (DY266) and human TNF DuoSet ELISA (DY210) from R&D Systems (Biotechne, Norway). Assays were performed as suggested by the manufacturer, and supernatants were stored at −80°C after collection and unfrozen on ice just before running the assays.

### 2.9 Statistical analysis

Data that were assumed to follow a log-normal distribution were log-transformed before statistical analysis. Quantification of gene expression by qRT-PCR was log-transformed and analyzed by repeated measures analysis of variance (RM-ANOVA) or a mixed model if there were missing data, followed by Holm–Šídák’s multiple comparisons post-test. ELISA and BioPlex data were analyzed using a Wilcoxon matched-pairs signed-rank test, a Mann–Whitney test, or a paired *t*-test. For the data from WB analysis, significance was evaluated by *t*-test with Welch’s correction. All graphs and analyses were generated with GraphPad Prism v10.1.0 (Dotmatics).

## 3 Results

### 3.1 HMPV induces *TNF* expression earlier than *IFNB*1 in THP−1 cells and human monocyte−derived macrophages

To determine the impact of TLR4 and SLAMF1 on HMPV-stimulated TNF and IFN-β responses in macrophages, we aimed to use THP-1 cells as a supporting model system to primary human monocyte-derived macrophages (MDMs), as THP-1 cells are more amenable to genetic manipulation (e.g., TLR4 and SLAMF1 knockout or overexpression studies). However, since transcriptional differences between THP-1 cells and MDMs have been reported (48, 49), we first compared HMPV-induced *TNF* and *IFNB1* mRNA expression in THP-1 cells and MDMs. Human MDMs were differentiated from peripheral blood monocytes of healthy donors for 7 days, while THP−1 cells were differentiated into macrophage−like cells using phorbol 12-myristate 13-acetate (PMA). Both cell types were infected with HMPV (1–20 h) or treated with LPS, and lysates were collected at defined time points for parallel RNA and protein analyses.

**Figure 1:**
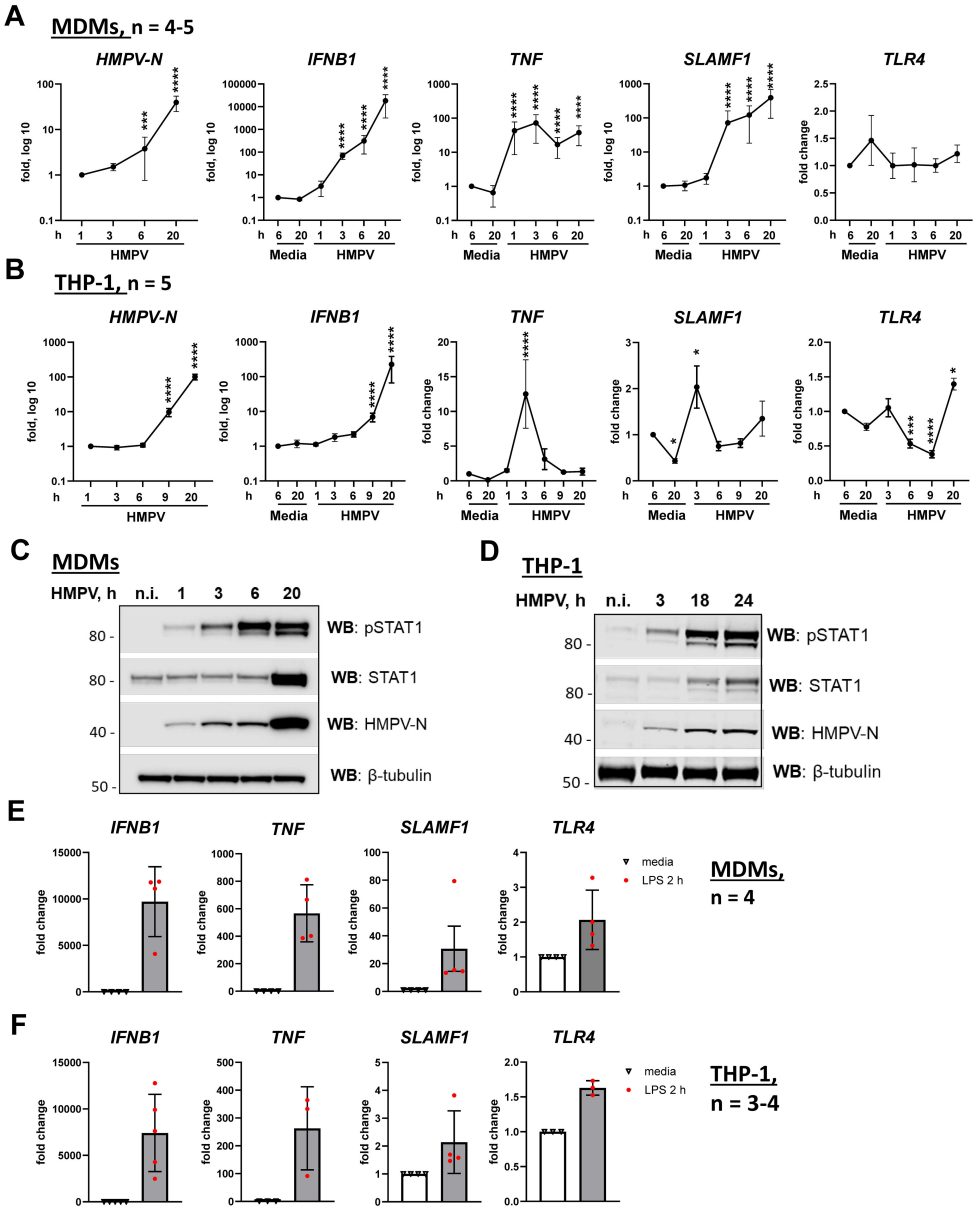
HMPV induces *TNF* mRNA expression at earlier time points of infection than *IFNB1* in both MDMs and THP-1 cells. **(A, B)***HMPV-N* vRNA expression and *IFNB1*, *TNF*, *SLAMF1*, and *TLR4* mRNA expression were evaluated by qRT-PCR in MDMs or THP-1 cells (*n* = 5) infected with HMPV (MOI = 1). Results were normalized to non-infected samples (media 6 h) or, for *HMPV-N* vRNA, to the level detected 1 h after infection. Data are presented as mean ± SD, with statistical testing (comparison with media 6-h values) performed by two-way ANOVA on log-transformed data (***p < 0.001, ****p < 0.0001). **(C, D)** Western blot (WB) analysis was performed to determine the total and phosphorylated (Tyr701) STAT1 and HMPV-N protein expression by MDMs **(C)** or THP-1 cells **(D)** at different time points of infection. Representative images are presented for one out of four individual donors **(C)** or one out of four experiments for THP-1 cells **(D)**. WB for β-tubulin was used as an endogenous loading control. **(E, F)***IFNB1*, *TNF*, and *SLAMF1* mRNA expression was evaluated by qRT-PCR in MDMs or THP-1 WT cells stimulated by LPS (100 ng/mL) for 2 h. **(E, F)** Data are presented as the mean of relative fold change ± SD.

The mRNA levels of *IFNB1*, *TNF*, viral nucleocapsid *N* (*HMPV-N*), *SLAMF1*, and *TLR4* were quantified by RT-qPCR (**Figures 1A, B, E, F**). In parallel, phosphorylated and total STAT1 protein, as well as HMPV-N protein, was assessed by Western blotting (**Figures 1C, D**). STAT1 total levels may be induced by type I IFNs also in HMPV-infected cells (16, 50, 51), and we therefore analyzed both phosphorylated and total STAT1 levels. In both MDMs and THP-1 cells, *HMPV-N* mRNA accumulated progressively over time, with a slight delay in THP-1 cells compared with MDMs (**Figures 1A, B**, first panels). HMPV infection induced a gradual increase in *IFNB1* expression in both cell types, with higher levels in MDMs than in THP-1 cells (**Figures 1A, B**, second panels). Consistently, phospho-STAT1 and total STAT1 protein levels increased toward the late stages of infection (20–24 h) in both cell types (**Figures 1C, D**), reflecting active type I IFN signaling (52).

Notably, *TNF* mRNA was strongly induced early during HMPV infection, peaking approximately 3 h in both MDMs and THP-1 cells (**Figures 1A, B**, third panels). While *TNF* levels subsequently declined in THP-1 cells between 6 and 20 h, MDMs maintained a higher expression during these later stages (**Figures 1A, B**). Strikingly, HMPV also induced *SLAMF1* expression, with a marked upregulation in MDMs and a more moderate response in THP-1 cells (**Figures 1A, B**, fourth panels). In both cell types, *SLAMF1* expression patterns paralleled those of *TNF*, with sustained induction in MDMs and a transient peak in THP-1 cells (**Figures 1A, B**, third and fourth panels).

To investigate whether modulation of TLR4 expression influences HMPV-induced responses, we examined *TLR4* mRNA levels and observed that, while *TLR4* expression in MDMs was not significantly affected by HMPV infection, *TLR4* mRNA in THP-1 cells was significantly reduced at 6 and 9 h post-infection and subsequently upregulated at 20 h (**Figures 1A, B**, right panels). The transient reduction in *TLR4* expression at 6 and 9 h in THP-1 cells could account, at least in part, for the more rapid decline in *TNF* mRNA induction observed in THP-1 cells compared with MDMs (**Figures 1A, B**, third panels). As expected and consistent with prior studies (53), *TLR4* mRNA was modestly increased in response to LPS (**Figures 1E, F**, last panels).

In summary, *TNF* was induced earlier than *IFNB1* following HMPV infection in both THP-1 cells and MDMs. However, when comparing THP-1 cells with MDMs, HMPV-mediated induction of *IFNB1*, *TNF*, and *SLAMF1* mRNAs was higher in MDMs than in THP-1 cells, and the induction of *TNF* and *SLAMF1* by HMPV declined more rapidly in THP-1 cells than in MDMs. Consistent with previous reports (48, 49), these findings underscore transcriptional differences between THP-1 cells and MDMs in their responses to inflammatory stimuli, demonstrating that during HMPV infection, particularly *SLAMF1* but also *TNF* and *IFNB1* are induced to higher levels in MDMs. Although these results highlight the limitations of using THP-1 cells as a standalone macrophage model, we consider THP-1 cells to represent a suitable supporting system to MDMs for investigating the roles of TLR4 and SLAMF1 in the regulation of *IFNB1* and *TNF* induction, as both cell types exhibited comparable patterns of early *TNF* and progressive *IFNB1* induction upon HMPV infection.

### 3.2 Knockout of *TLR4* reduces HMPV−induced *TNF* expression without affecting *IFNB1*

**Figure 2:**
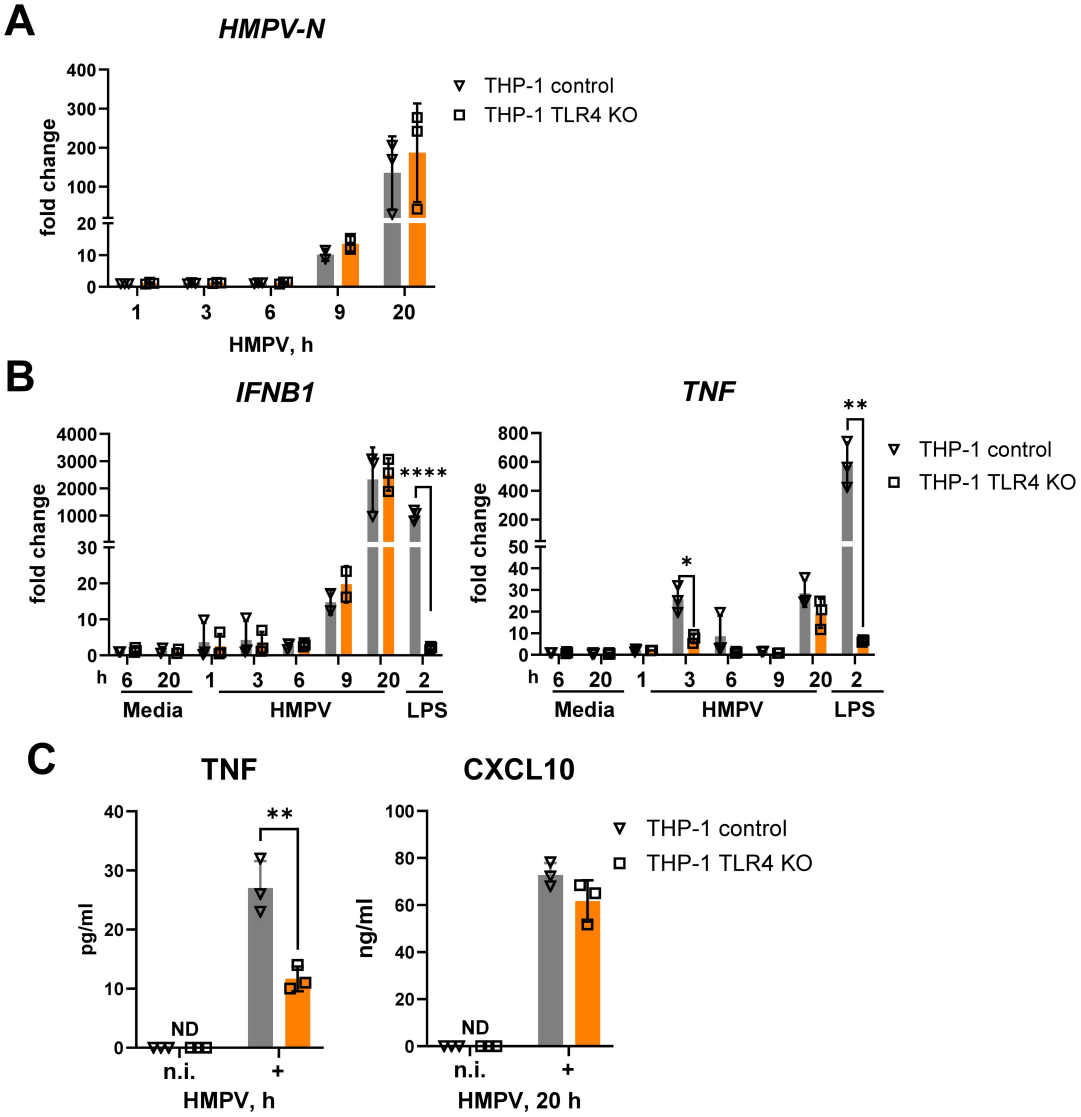
*TLR4* knockout in THP-1 cells strongly reduces the expression of HMPV-mediated TNF mRNA and protein without affecting *IFNB1* mRNA expression and IFN-β*-*dependent CXCL10 secretion. *HMPV-N* vRNA expression **(A)** and *IFNB1* and *TNF* mRNA expression **(B)** were determined by qRT-PCR in THP-1 control cells and THP-1 TLR4 KO cells infected with HMPV (MOI = 1) for the indicated time points or stimulated by LPS for 2 h (*n* = 3). Results were normalized to non-infected (n.i.) samples. Data are presented as mean ± SD, with statistical testing performed by two-way ANOVA on log-transformed data (*p < 0.05, ****p < 0.0001). **(C)** TNF and CXCL10 secretion was determined by ELISA of supernatants from non-infected (n.i.) or HMPV-infected cells (20 h), and data are presented as mean ± SD (*n* = 3). Statistical significance was assessed using a paired *t*-test, and only significant results are indicated (**p < 0.01; ND, not detected).

TLR4 regulates the induction of both *TNF* and *IFNB1* in response to LPS through different signaling complexes formed with TIRAP/MyD88 at the plasma membrane and TRAM/TRIF following endocytosis (12). To evaluate if TLR4 contributes to *TNF* and *IFNB1* expression during HMPV infection, we next made use of a THP-1 TLR4 knockout subline (THP-1 TLR4 KO) alongside control THP-1 cells expressing non-targeting guide RNA. Both cell types were infected with HMPV for various time points, followed by analysis of *HMPV-N* (**Figure 2A**), *TNF*, and *IFNB1* mRNA levels (**Figure 2B**) and measurement of secreted TNF and CXCL10 (**Figure 2C**). CXCL10 can be induced not only by type I interferons but also by other cytokines (54–57). To assess the contribution of type I IFN signaling to HMPV-induced *CXCL10* expression, we examined the effect of anti-IFNAR neutralizing antibodies. Pretreatment with anti-IFNAR antibodies almost completely abrogated HMPV-induced *CXCL10* expression (**Supplementary Figure S1**), indicating that *CXCL10* induction is largely dependent on type I IFN signaling and thus reflects the levels of secreted type I IFNs. Our results showed that secreted TNF, but not CXCL10, was reduced in HMPV-infected THP-1 TLR4 KO cells (**Figure 2C**) and confirmed that TLR4 knockout did not impair HMPV-induced *IFNB1* levels. *HMPV-N* mRNA expression was comparable between the control and TLR4 KO cells (**Figure 2A**), indicating that differences in cytokine expression were not due to altered viral replication.

TLR4 deficiency markedly reduced the early (3 h post-infection) HMPV-induced *TNF* mRNA expression, whereas HMPV-stimulated *IFNB1* mRNA expression was unaffected (**Figure 2B**). As expected, TLR4 knockout abolished LPS-induced expression of both *TNF* and *IFNB1* (**Figure 2B**). The selective effect of TLR4 knockout on HMPV-mediated *TNF* was further reflected at the protein level: secreted TNF was significantly reduced at 20 h post-infection in TLR4 KO cells, while secretion of the IFN-inducible chemokine CXCL10 remained unchanged (**Figure 2C**).

Together, these results show that in THP-1 cells, TLR4 is required for early HMPV-induced *TNF* expression but does not regulate HMPV-induced *IFNB1*.

### 3.3 TLR4 overexpression enhances HMPV−induced p38 MAPK activation and *TNF* and *IFNB1* expression in THP−1 cells

**Figure 3:**
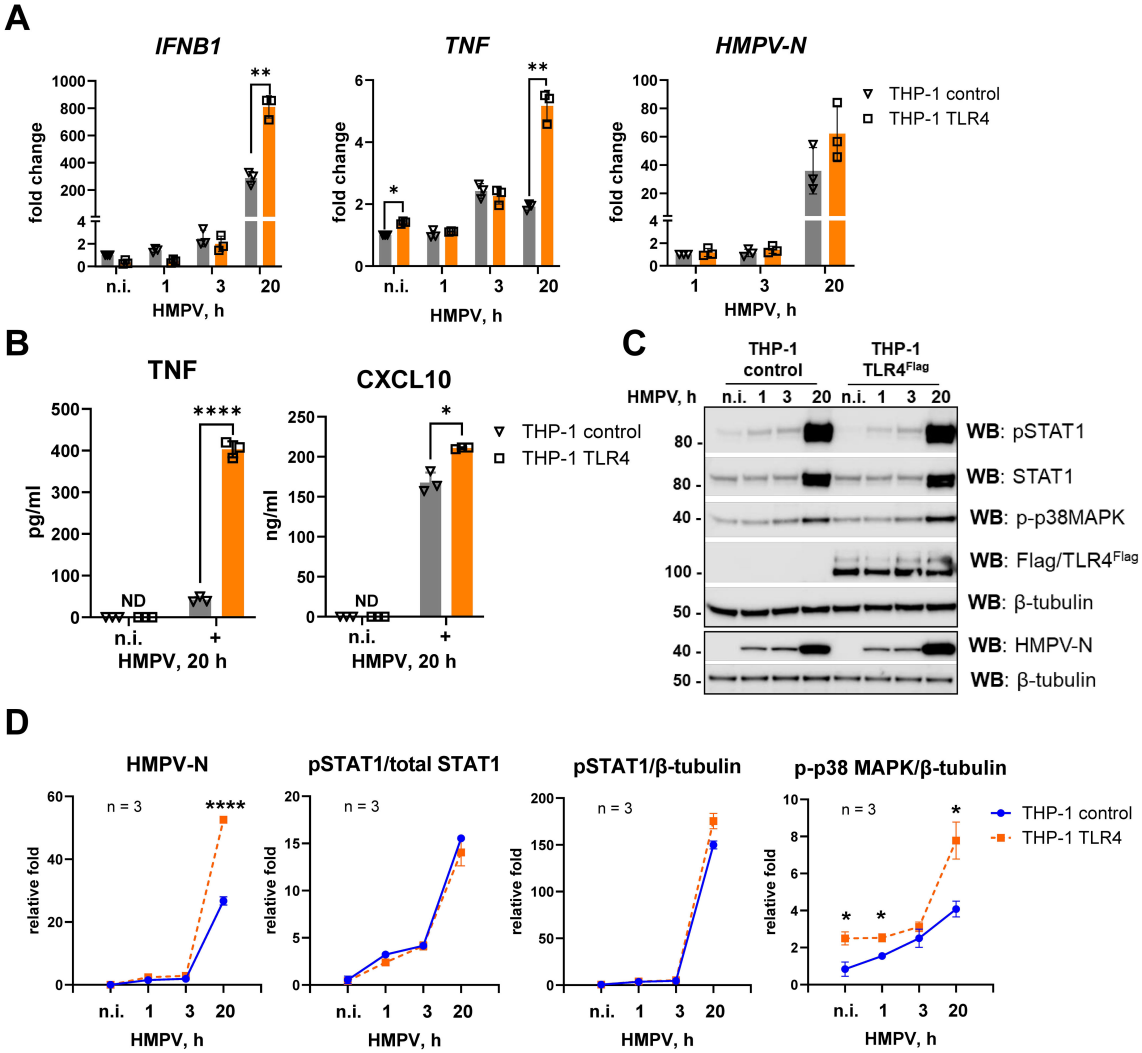
TLR4 overexpression in THP-1 cells enhances HMPV-stimulated *TNF* and *IFNB1* induction and p38 MAPK activation. **(A)***HMPV-N* vRNA expression and *IFNB1* and *TNF* mRNA expression were determined by qRT-PCR in THP-1 control cells and THP-1 TLR4^Flag^ cells infected by HMPV (MOI = 1) for the indicated time points or stimulated by LPS for 2 h (*n* = 3). The results were normalized to non-infected (n.i.) samples or, for *HMPV-N*, to the level detected 1 h after infection in control cells. Statistical testing was done by two-way ANOVA on log-transformed data (**p < 0.01). **(B)** TNF and CXCL10 cytokine secretion levels were determined by ELISA of supernatants from non-infected (n.i.) or HMPV-infected cells (20 h), and data are presented as mean ± SD (*n* = 3). Statistical significance was evaluated by paired *t*-test; significance level: *p < 0.05, ****p < 0.0001; ND, not detected. **(C)** WB analysis was performed to determine the expression of total and phosphorylated (Tyr701) STAT1, phospho-p38MAPK (Thr180/Tyr182), HMPV-N, and TLR4^Flag^ in THP-1 sublines at different time points of HMPV infection (a representative image is shown, *n* = 4). WB for β-tubulin was used as an endogenous control. **(D)** Graphs show combined data for three experiments (analyzed in LiCor Odyssey software) of HMPV-N, pSTAT1, or p-p38 MAPK protein levels relative to β-tubulin or pSTAT1 relative to total STAT1. Statistical significance was assessed using a *t*-test with Welch’s correction, and only significant results are indicated (*p < 0.05, **p < 0.01).

We next examined how TLR4 overexpression affected HMPV-induced *IFNB1* and *TNF* expression by comparing a previously generated (46) THP-1 subline overexpressing TLR4-Flag (THP-1 TLR4) with control THP-1 cells carrying an empty vector. TLR4 overexpression significantly increased HMPV-stimulated *TNF* and *IFNB1* mRNA levels at later stages of infection (**Figure 3A**). Although HMPV-stimulated *IFNB1* induction was not reduced in TLR4-deficient THP-1 cells (**Figure 2B**), overexpression of TLR4 enhanced *IFNB1* expression (**Figure 3A**). The reason for this apparent discrepancy remains unclear; however, it is possible that other PRRs, such as DC-SIGN, may compensate for the absence of TLR4 in HMPV-mediated *IFNB1* induction, as has been reported for other pathogen–PRR interactions (58, 59).

*HMPV-N* mRNA and protein levels were also elevated in TLR4-overexpressing cells at 20 h post-infection (**Figures 3A, C, D**). In line with the mRNA data, secretion of TNF and CXCL10 was increased in TLR4-overexpressing THP-1 cells compared with controls (**Figure 3B**). In addition, phosphorylated STAT1, which we have previously shown to depend strongly on IFN receptor signaling in HMPV-infected MDMs (16), was modestly increased in TLR4-overexpressing cells (**Figures 3C, D**).

Overexpression of TLR4 in THP-1 cells was confirmed by immunoblotting (**Figure 3C**), and its functional activity was validated by enhanced induction of *IFNB1* and *TNF* mRNA in response to LPS stimulation (**Supplementary Figure S2**).

The p38 MAPK pathway, known to be activated downstream of multiple PRRs and to regulate TLR4-mediated *TNF* expression in response to LPS (60, 61), had not been directly examined in the context of HMPV infection. Interestingly, we observed that HMPV triggered p38 MAPK phosphorylation, and this activation was significantly increased in TLR4-overexpressing THP-1 cells compared with control cells (**Figures 3C, D**).

Collectively, these findings indicate that TLR4 overexpression in THP-1 cells enhances HMPV-induced p38 MAPK activation and promotes increased *TNF* and *IFNB1* expression in THP-1 cells.

### 3.4 SLAMF1 overexpression enhances HMPV−induced p38 MAPK activation and *TNF* secretion

In human macrophages, we have previously shown that SLAMF1 is critical for *E. coli*- and LPS-TLR4-mediated *IFNB1* expression, while its effect on *TNF* expression was more modest (38). To investigate whether SLAMF1 influences HMPV-induced *IFNB1* and *TNF* responses, we generated THP-1 cells overexpressing SLAMF1 (THP-1 SLAMF1) along with control cells with an empty vector (THP-1 control). SLAMF1 overexpression was confirmed by Western blotting (**Supplementary Figure S3**).

**Figure 4:**
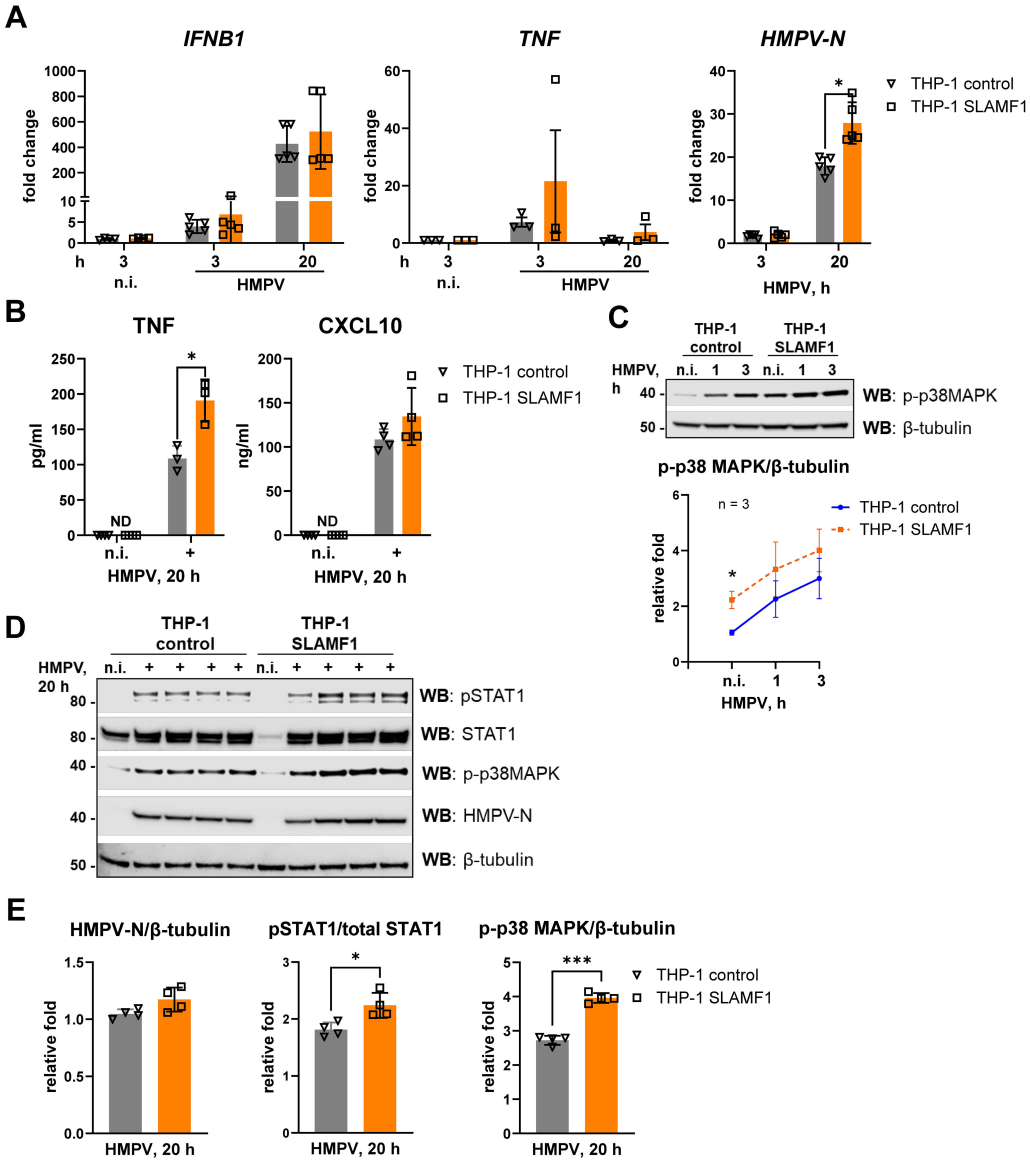
SLAMF1 overexpression in THP-1 cells increases HMPV-stimulated p38 MAPK activation and TNF secretion. **(A)***IFNB1*, *TNF*, and *HMPV-N* vRNA mRNA expression was determined by qRT-PCR analysis of THP-1 control cells and THP-1 SLAMF1 cells infected with HMPV (MOI = 1) for the indicated time points (*n* = 3–5). Results were normalized to non-infected (n.i.) samples or, for *HMPV-N*, to the level detected 3 h after infection in control cells. Statistical testing was done by two-way ANOVA on log-transformed data (**p < 0.01). **(B)** TNF and CXCL10 cytokine secretion levels were determined by ELISA of supernatants from non-infected (n.i.) or HMPV-infected cells (20 h), and the data are presented as mean ± SD (*n* = 3–4). Statistical significance was evaluated paired *t*-test, with no significant results found; ND, not detected. **(C, D)** WB was performed to determine **(C)** the expression of phospho-p38MAPK (Thr180/Tyr182) at early time points of HMPV infection (representative image, *n* = 3) and **(D, E)** the expression of total and phosphorylated (Tyr701) STAT1, phospho-p38MAPK, and HMPV-N (results of four independent experiments are shown together on the blots, 20 h of infection) in THP-1 sublines. **(C, D)** β-Tubulin was used as an endogenous control. **(E)** Quantification of HMPV-N or p-p38 MAPK protein levels relative to β-tubulin or pSTAT1 relative to total STAT1 was performed using LiCor Odyssey software, combining data for the four experiments. Statistical significance was assessed using a paired *t*-test, and only significant results are indicated (*p < 0.05, **p < 0.01).

HMPV-stimulated *IFNB1* mRNA levels and secretion of the IFN-inducible protein CXCL10 were largely unaffected by SLAMF1 overexpression in THP-1 cells. In contrast, *TNF* mRNA expression and TNF secretion were significantly increased in SLAMF1-overexpressing THP-1 cells (**Figures 4A, B**). Notably, *TNF* mRNA induction was enhanced as early as 3 h post-infection, with a corresponding increase in secreted TNF observed after 20 h of HMPV infection (**Figures 4A, B**). Additionally, *HMPV-N* mRNA levels were significantly higher in SLAMF1-overexpressing THP-1 cells at 20 h post-infection (**Figure 4A**).

We have previously demonstrated that SLAMF1 is required for TLR4-driven activation of p38 MAPK (38). Consistent with this, SLAMF1 overexpression significantly enhanced HMPV-induced phosphorylation of p38 MAPK (**Figures 4C–E**), similar to the increased p38 MAPK activation observed in TLR4-overexpressing cells (**Figure 3C**). In agreement with the observation that SLAMF1 overexpression did not markedly increase HMPV-induced *IFNB1* or CXCL10, phosphorylation of STAT1 by HMPV was modestly elevated in SLAMF1-overexpressing cells (**Figures 4D, E**). The reason why we do not observe a directly correlated output between *IFNB1* levels and phosphorylated STAT1 may relate to different optimal kinetics of these responses and the experimental time points used for analysis.

Taken together, these findings show that SLAMF1 overexpression in THP-1 cells enhances HMPV-triggered p38 MAPK activation and *TNF* induction, while having only a limited effect on HMPV-induced *IFNB1* expression in THP-1 cells.

### 3.5 *TLR4* and *SLAMF1* silencing in human MDMs reduces HMPV-induced *TNF* expression

While we used THP-1 to pinpoint effects of overexpression and knockout of SLAMF1 and TLR4 on *TNF* and *IFNB1* induction, THP-1 showed lower HMPV-stimulated induction of *SLAMF1*, *TNF*, and *IFNB1* and had been shown to be phenotypically different from MDMs (48, 49). Hence, for translational significance, it is of utmost importance to validate findings from THP-1 cells in human primary MDMs. Hence, we next examined the effects of TLR4 and SLAMF1 silencing on HMPV-induced *TNF* and *IFNB1* expression in human primary MDMs derived from healthy donors (*n* = 6). As we find that commercially available antibodies for TLR4 and SLAMF1 have low specificity in immunoblotting, efficient silencing was confirmed by significantly reducing *TLR4* and *SLAMF1* mRNA levels at all tested infection time points, as well as reducing LPS-induced *TNF* and *IFNB1* in siTLR4-treated MDMs (**Supplementary Figure S4**). The LPS-mediated cytokine response is completely dependent on TLR4 expression and could serve as an additional readout for TLR4 expression (62). The efficiency of our *SLAMF1* siRNA in MDMs has previously been validated by confocal microscopy (38).

**Figure 5:**
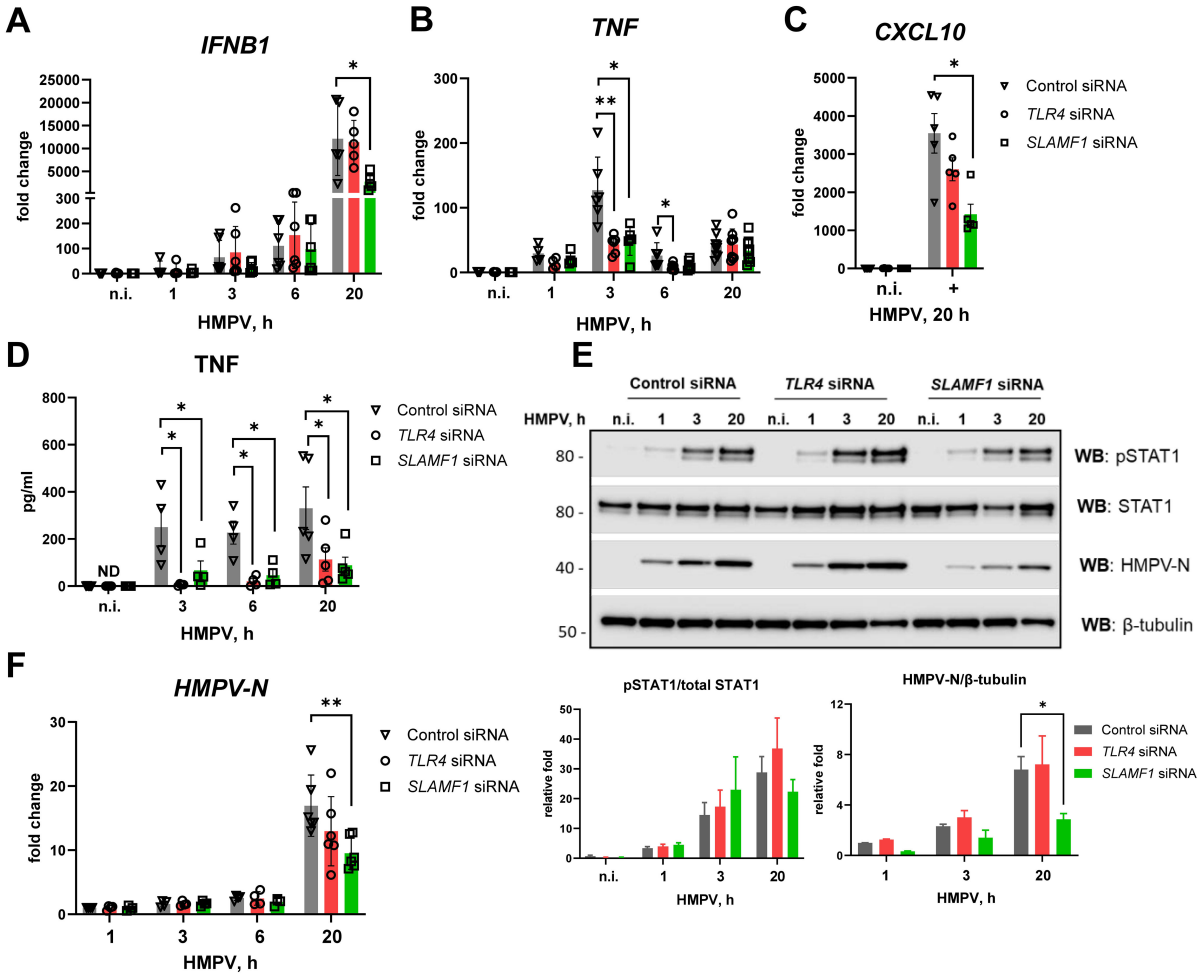
*TLR4* or *SLAMF1* silencing in human MDMs reduces HMPV-mediated *TNF* expression, while only SLAMF1 silencing decreases *IFNB1* induction. Primary human MDMs were transfected with control, *TLR4*, and *SLAMF1* siRNA oligos and infected with HMPV for the indicated time points (*n* = 5–8). **(A, B)** Expression of *IFNB1***(A)**, *TNF***(B)**, and *CXCL10***(C)** mRNA was evaluated by qRT-PCR, and the results were normalized to non-infected (n.i.) samples. **(D)** TNF cytokine secretion level was determined by ELISA of supernatants from non-infected (n.i.) or HMPV-infected cells (20 h). **(E)** WB analysis (representative image, *n* = 4) was performed to determine total and phosphorylated (Tyr701) STAT1 levels and HMPV-N protein expression. β-Tubulin was used as an endogenous control for loading. Graphs show combined data for quantification of HMPV-N protein level relative to β-tubulin or pSTAT1 relative to total STAT1 (LiCor Odyssey software). **(F)***HMPV-N* vRNA expression was determined by qRT-PCR, and the results were normalized to the level detected after 1 h of infection of control cells. Data on graphs are presented as mean of relative fold change ± SEM **(A–D, F)** or ± SD **(E)**. Statistical testing was done by two-way ANOVA on log-transformed data **(A–C, F)** or by a multiple Wilcoxon test **(D)** or by a paired *t*-test **(E)**, and only significant results are indicated (*p < 0.05, **p < 0.01; ND, not detected).

Silencing of *SLAMF1* or *TLR4* had minimal impact on HMPV-induced *IFNB1* mRNA expression, except for a reduction observed in *SLAMF1*-silenced MDMs at 20 h post-infection (**Figure 5A**). In contrast, HMPV-induced *TNF* mRNA expression in MDMs was significantly reduced at 3 h post-infection following silencing of either *SLAMF1* or *TLR4* (**Figure 5B**). To evaluate the secreted type I IFN levels in *SLAMF1*- and *TLR4*-silenced MDMs after 20 h of HMPV infection (**Figure 5A**), we assessed *CXCL10* expression, which critically depends on type I IFN signaling in HMPV-infected MDMs (**Supplementary Figure 1**). Consistent with the reduced *IFNB1* mRNA levels in *SLAMF1*-silenced MDMs, *CXCL10* induction was significantly decreased upon *SLAMF1* depletion (**Figure 5C**), indicating that secreted type I IFNs are indeed reduced in HMPV-infected *SLAMF1*-depleted MDMs. Similarly, in line with the reduced *TNF* mRNA induction, TNF secretion was markedly diminished in *TLR4*- or *SLAMF1*-depleted MDMs across all examined time points (**Figure 5D**).

Knockdown of *TLR4* or *SLAMF1* did not substantially alter HMPV-induced STAT1 phosphorylation at early time points, although SLAMF1 depletion significantly reduced STAT1 phosphorylation at 20 h post-infection (**Figure 5E**), consistent with the reduced *HMPV-N* mRNA and protein levels in these MDMs (**Figures 5E, F**). Comparing these results to THP-1 cells, we note that SLAMF1 affected late IFNB1 induction in MDMs but did not affect IFNB1 induction in THP-1 cells (**Figure 4**), suggesting that SLAMF1 may be expressed at higher levels in HMPV-infected MDMs (**Figure 1**) and hence impact IFNB1 expression in human MDMs.

Collectively, these results indicate that both TLR4 and SLAMF1 are required for the early induction of *TNF* mRNA by HMPV in human MDMs, while only SLAMF1 contributes to HMPV-mediated *IFNB1* expression at later stages of infection.

### 3.6 HMPV−induced p38 MAPK activation is regulated by TLR4 and SLAMF1 and drives *TNF* and *IFNB1* induction in human macrophages

**Figure 6:**
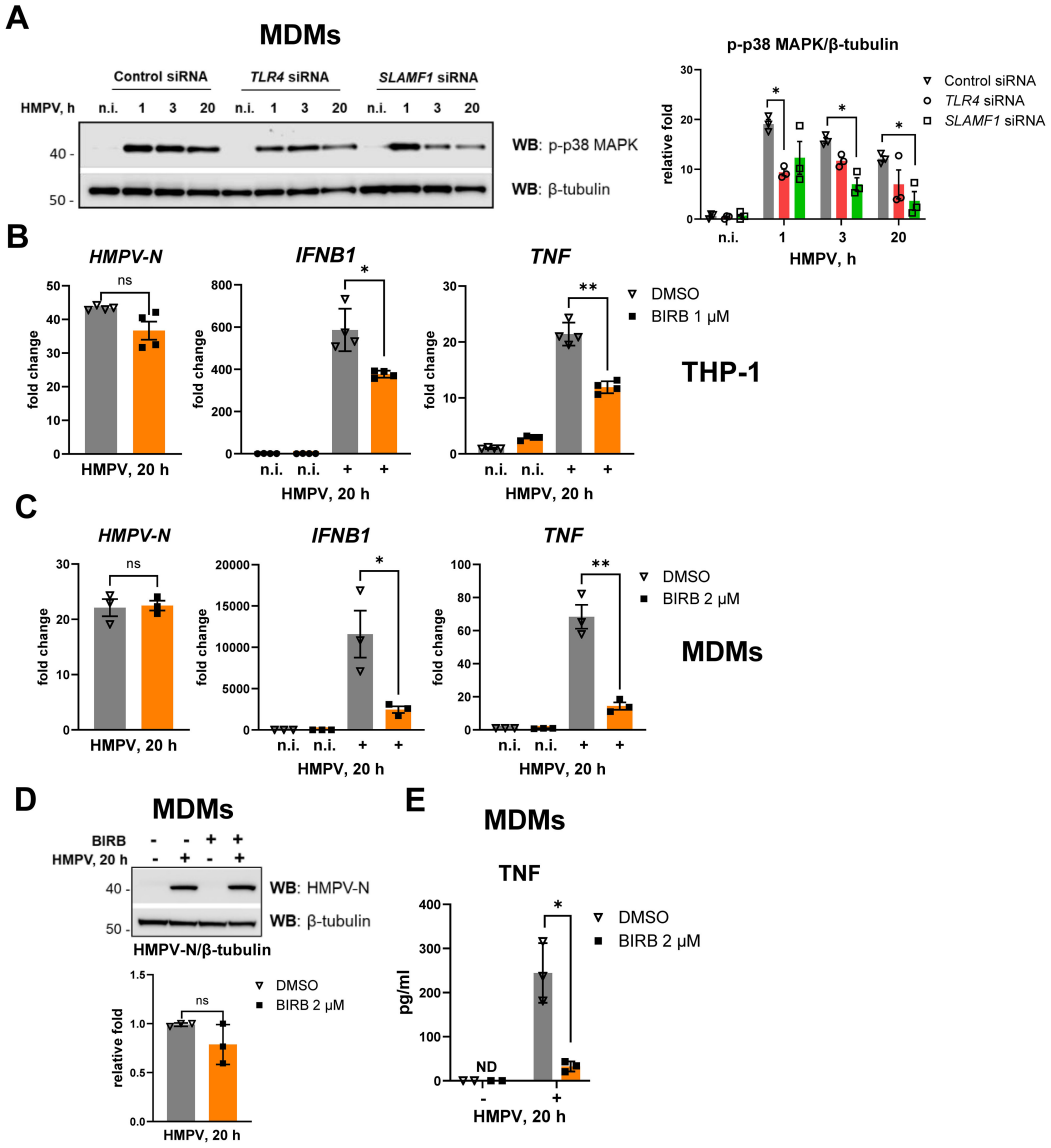
Silencing of *SLAMF1* or *TLR4* inhibits HMPV-mediated phosphorylation of p38 MAPK, and p38 MAPK inhibition decreases HMPV-induced *IFNB1* and *TNF* expression. **(A)** WB analysis of phospho-p38 MAPK (Thr180/Tyr182) in lysates of primary human MDMs transfected with siRNA oligos (control, *TLR4*, and *SLAMF1* siRNA) and infected with HMPV (representative image, *n* = 3). The graph shows combined data for all the experiments with phospho-p38 MAPK levels normalized to β-tubulin (loading control). **(B, C)***HMPV-N* vRNA expression and *IFNB1* and *TNF* mRNA expression were determined by qRT-PCR in THP-1 cells or primary human MDMs pretreated with DMSO (control) or the BIRB796 p38 MAPK inhibitor for 30 min before HMPV infection (MOI = 1) for 20 h (*n* = 3). Results were normalized to non-infected (n.i.) samples and for *HMPV-N* to the level detected 1 h after infection in DMSO-treated cells. Data are presented as mean relative fold change ± SD (THP-1 cells, **B**) or ± SEM (MDMs, **C**). Statistical testing was done by unpaired *t*-test (ns, not significant; *p < 0.05, **p < 0.01). **(D)** WB analysis was performed to determine HMPV-N protein levels in MDMs pretreated with DMSO or BIRB796 and infected with HMPV for 20 h (*n* = 3). The graph represents combined data for all experiments, normalized to β-tubulin (loading control). Statistical significance was evaluated using a paired *t*-test; significance level (ns, not significant). **(E)** TNF secretion levels were examined by ELISA in the supernatants from non-infected (n.i.) or HMPV-infected cells (20 h), with data presented as mean ± SD (*n* = 3); ND, not detected. Statistical significance was evaluated using a paired *t*-test, and only significant results are indicated (*p < 0.05).

We observed that HMPV infection induces p38 MAPK phosphorylation in THP-1 cells, and this effect is enhanced by TLR4 and SLAMF1 (**Figures 3**, **4**). To further assess the contribution of these receptors to HMPV-mediated p38 MAPK activation in human primary MDMs, we examined p38 MAPK phosphorylation in MDMs transfected with *TLR4* or *SLAMF1* siRNAs and subsequently infected with HMPV at different time points. HMPV infection triggered robust p38 MAPK phosphorylation in MDMs as early as 1 h post-infection, with activation slightly reduced but maintained throughout 20 h (**Figure 6A**). Silencing either *TLR4* or *SLAMF1* reduced HMPV-induced p38 MAPK phosphorylation from the earliest analyzed time point (**Figure 6A**), indicating that both receptors contribute to early p38 MAPK activation in MDMs, possibly through the TLR4–TIRAP–MyD88 signaling axis, which is positively regulated by SLAMF1 in human macrophages (38).

To evaluate the functional role of p38 MAPK activation in HMPV-induced cytokine expression, THP-1 cells and MDMs were pretreated with the p38 MAPK inhibitor BIRB796 prior to HMPV infection for 20 h. Inhibition of p38 MAPK significantly reduced the induction of *TNF* and *IFNB1* mRNAs in HMPV-infected THP-1 cells (**Figure 6B**) and macrophages (**Figure 6C**), while *HMPV-N* RNA (**Figures 6B, C**) and protein levels (**Figure 6D**) remained unchanged. Consistent with the reduced *TNF* mRNA, TNF secretion was strongly impaired by p38 MAPK inhibition in MDMs (**Figure 6E**). The cytotoxicity of BIRB796 at the concentrations used in **Figures 6B–E** (1–2 μM) was assessed by LDH release assay, and no cytotoxic effects were detected (**Supplementary Figure S5A**). To evaluate the specificity of BIRB796, given that it may partially inhibit JNKs (63), we examined the effects of the additional p38 MAPK inhibitors SB202190 and SB203580 on HMPV-induced *IFNB1* and *TNF* expression (**Supplementary Figure S5B**). SB202190 and SB203580 specifically inhibit p38 MAPK activity without affecting JNK activity (64–67). Both inhibitors reduced HMPV-induced *IFNB1* and *TNF* expression to a similar extent as BIRB796 (**Supplementary Figure S5B**). Finally, we confirmed that BIRB796 abrogated the phosphorylation of the downstream p38 MAPK target MK2/MAPKAPK2 while having no effect on the phosphorylation of JNKs or the JNK target protein ATF2 (**Supplementary Figure S5C**). Collectively, these findings indicate that the inhibitory effect of BIRB796 on HMPV-induced *IFNB1* and *TNF* expression results from specific inhibition of p38 MAPK signaling.

Collectively, these findings demonstrate that in THP-1 cells and human MDMs, TLR4 and SLAMF1 contribute to HMPV-mediated p38 MAPK activation, leading to TNF expression, and that p38 MAPK contributes to *IFNB1* induction in both THP-1 cells and MDMs.

## 4 Discussion

In this study, we identify TLR4 and SLAMF1 as immune receptors that regulate the expression of the pro-inflammatory cytokine TNF during HMPV infection in human primary macrophages. Both TLR4 and SLAMF1 contribute to activation of the p38 MAPK pathway, which we show is an important signaling step for HMPV-mediated *TNF* induction.

Understanding the mechanisms controlling TNF expression during viral infection is critical, as this cytokine has been linked to severe disease caused by major respiratory viruses such as SARS-CoV-2, influenza virus, and the pneumoviruses RSV and HMPV (68–70). Moreover, TNF is a principal mediator of RSV-induced disease, and elevated nasal TNF levels correlate with severity in infants with RSV bronchiolitis (70). For HMPV, we and others (24, 28) have reported that higher TNF levels in nasopharyngeal aspirates and blood are associated with pneumonia and bronchiolitis severity, supporting a critical role for TNF in HMPV-induced lung pathology. While TNF contributes to viral clearance early in infection, as shown for influenza virus and RSV (71, 72), excessive TNF, together with IFN-γ, can drive cell death, tissue damage, and mortality, as demonstrated in SARS-CoV-2 infection (68). The involvement of a TLR4–SLAMF1–p38 MAPK axis in early TNF induction by HMPV has not been reported and could aid therapeutic options to regulate TNF levels.

In this study, we employed THP-1 cells as a supporting system to MDMs, as THP-1 cells are more amenable to genetic manipulation. Notably, HMPV infection induced higher levels of *IFNB1*, *TNF*, and *SLAMF1* expression in MDMs than in THP-1 cells. Moreover, *TNF* induction declined more rapidly in THP-1 cells. These observations are consistent with previous reports demonstrating transcriptional differences between MDMs and THP-1 cells (73) and underscore the importance of validating findings obtained in THP-1 cells using primary macrophages.

**Figure 7:**
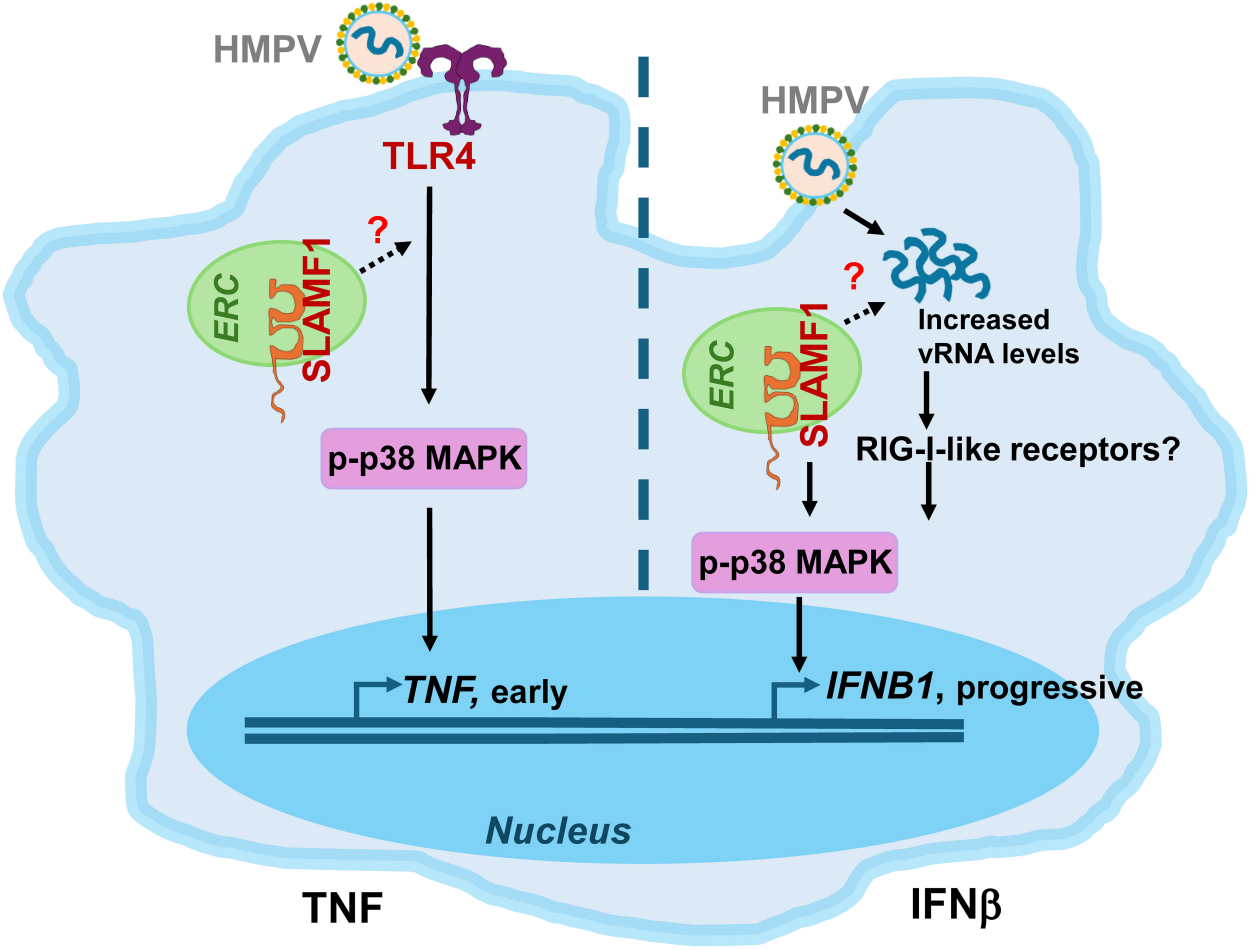
Proposed model of *TNF* and *IFNB1* induction by HMPV. SLAMF1 is localized in the endocytic recycling compartment (ERC) in unstimulated human MDMs (38). *Left panel, TNF induction*: SLAMF1 and TLR4 stimulate early p38 MAPK phosphorylation, driving early TNF induction in both THP-1 cells and MDMs. SLAMF1 could facilitate TLR4-mediated signaling to p38 MAPK-TNF induction by ERC-mediated trafficking mechanisms, similar to that previously shown for LPS-driven p38 MAPK-TNF induction in primary human macrophages (38). SLAMF1 enhances p38 MAPK-TNF in both THP-1 cells and MDMs. *Right panel, IFNB1* induction: HMPV infection and increased HMPV vRNA levels enhance p38 MAPK-regulated *IFNB1* induction in both THP-1 cells and MDMs, potentially via RIG-I-like receptors. SLAMF1 could contribute to the increased vRNA levels and IFNB1 induction and appears to play a more prominent role in human MDMs than in THP-1 cells. HMPV, human metapneumovirus; TLR4, Toll-like receptor 4; vRNA, viral RNA; RIG-I-like receptors, retinoic acid-inducible gene-I-like receptors; MAPK, mitogen-activated protein kinase.

Our results further show that HMPV triggers *TNF* and *IFNB1* expression with distinct kinetics in both MDMs and THP-1 cells: *TNF* peaks early, whereas *IFNB1* gradually accumulates over time. This suggests that different receptors mediate these responses. Indeed, we found that TLR4 overexpression in THP-1 cells, or conversely, TLR4 depletion in THP-1 cells or MDMs, respectively, enhanced or reduced *TNF* induction by HMPV, while the effects on *IFNB1* expression varied depending on whether TLR4 was overexpressed or silenced. TLR4 overexpression in THP-1 cells enhanced HMPV-induced *IFNB1* expression, whereas TLR4 knockdown in THP-1 cells or MDMs did not significantly affect *IFNB1* levels. The basis for this discrepancy remains unclear, but it is possible that alternative PRRs, such as DC-SIGN, may compensate for the absence of TLR4 in HMPV-mediated *IFNB1* induction, as reported for other pathogen–PRR interactions (58, 59). Nevertheless, in both THP-1 cells and MDMs, TLR4 expression levels exerted a more pronounced effect on *TNF* induction than on *IFNB1* expression. This aligns with previous work showing that cytoplasmic RIG-I-like receptors are major drivers of IFN-β in macrophages and dendritic cells infected with HMPV and other RNA viruses (74–76). We therefore propose that TLR4 (together with SLAMF1) predominantly regulates early *TNF* induction, whereas RIG-I-like receptors contribute to progressive IFN-β production as viral RNA and replication intermediates accumulate (**Figure 7**).

Our findings also reinforce the emerging role of TLR4 in recognizing viral components. Several viral glycoproteins, including those from SARS-CoV-2, RSV, Ebola virus, and dengue virus, activate TLR4-dependent signaling (29, 31, 77, 78). For HMPV, the fusion protein was shown to be a potent TLR4 agonist that elicits inflammatory responses in monocytes, while the HMPV surface glycoprotein G modulated cytokine induction in dendritic cells via TLR4 signaling (10, 79). Although the clinical relevance of TLR4-mediated TNF production remains incompletely defined, TLR4 knockout mice show reduced manifestations of HMPV disease (11), and TLR4 polymorphisms that are associated with reduced function correlate with enhanced RSV disease susceptibility (80). Furthermore, the failure of a formalin-inactivated RSV vaccine candidate was linked to insufficient TLR4 activation, whereas the addition of TLR4 agonists improved its protection in animal models (78, 81).

In this study, we found that in HMPV-infected human macrophages, silencing of both *TLR4* and *SLAMF1* reduced *TNF* expression. We have previously shown that SLAMF1 enhances TLR4-mediated TNF expression and secretion by *E. coli* and LPS in human macrophages (38). Accordingly, the reduced levels of HMPV-induced TNF expression and secretion in SLAMF1-silenced MDMs could be explained by the regulatory role of SLAMF1 in TLR4-mediated signaling. Mechanistically, we speculate that SLAMF1, which we have previously shown to be localized in the endocytic recycling compartment (ERC) in human macrophages (38), enhances TLR4 signaling in response to HMPV, hence stimulating early p38 MAPK activation and subsequent *TNF* induction (schematically depicted in **Figure 7**). This is based on our current observation that SLAMF1 overexpression in THP-1 cells amplified HMPV-induced p38 activation and *TNF* output, that p38 and TNF secretion in MDMs was reduced by SLAMF1 silencing, and our previous findings showing that SLAMF1 overexpression increases TLR4-mediated p38 MAPK phosphorylation (38). Moreover, p38 MAPK activity was required for both *TNF* and *IFNB1* induction by HMPV (**Figures 6B, C**). However, although SLAMF1 overexpression in THP-1 cells enhanced p38 MAPK-dependent *TNF* induction (**Figure 4**), it did not significantly increase *IFNB1* expression in HMPV-infected THP-1 cells (**Figure 4**). We propose that the SLAMF1–p38 axis exerts a stronger influence on *TNF* than on *IFNB1*, as p38 MAPK is a key component of a positive feedback loop to *TNF* induction involving TNF signaling (82). In contrast, *IFNB1* induction primarily depends on interferon regulatory factors, with p38 MAPK acting in a modulatory capacity, as demonstrated for the RNA virus—Sendai virus (82–84).

When comparing THP-1 cells and MDMs, we found that the SLAMF1–p38 MAPK axis enhanced *TNF* induction in both models. Conversely, HMPV-induced p38 MAPK phosphorylation, *IFNB1*, and *CXCL10* expression were significantly reduced in siSLAMF1-treated MDMs (**Figures 5**, **6**), whereas SLAMF1 overexpression in THP-1 cells did not significantly enhance *IFNB1* or *CXCL10* secretion (**Figure 4**). These findings may reflect inherent differences in SLAMF1 function between MDMs and THP-1 cells.

We further observed that STAT1 phosphorylation in HMPV-infected macrophages peaks at later time points (20–24 h), whereas p38 MAPK phosphorylation occurs as early as 1 h post-infection and remains sustained, both in MDMs and THP-1 cells. To our knowledge, p38 MAPK involvement in HMPV-mediated cytokine induction has not been previously described. Here, silencing TLR4 or SLAMF1 in MDMs reduced HMPV-induced p38 MAPK activation at early time points, indicating that HMPV triggers signaling downstream of both receptors early during infection in MDMs. Inhibition of p38 MAPK markedly reduced both *TNF* and *IFNB1* induction, both in MDMs and in THP-1 cells (**Figure 6**), suggesting that p38 MAPK activity is required not only for early TLR4-driven *TNF* induction but may also contribute to IFN-β production downstream of RIG-I signaling at later stages, consistent with the findings for the Sendai virus (85).

Notably, at later time points of HMPV infection (20 h), *SLAMF1* silencing in human MDMs also reduced *IFNB1* expression, whereas *TLR4* silencing did not. Also, upon SLAMF1 depletion, we observed reduced HMPV-N mRNA and protein levels at late time points of infection, which could suggest that SLAMF1 affects viral uptake or replication that could lead to altered *IFNB1* expression. Indeed, SLAMF1 has been proposed as a measles virus receptor (86, 87) and has been implicated in virus uptake mechanisms such as macropinocytosis in immune cells (88). Although we did not address HMPV entry in this study, the decrease in HMPV levels after *SLAMF1* knockdown warrants further investigation.

Overall, our results identify distinct regulatory mechanisms for HMPV−mediated TNF and IFN-β in human macrophages, with TLR4 and SLAMF1 driving early p38 MAPK activation and TNF induction, while SLAMF1 also influences late IFN-β responses in MDMs. As suggested for SARS−CoV−2 and RSV, therapeutic modulation of TNF−mediated inflammation could be a promising strategy to limit tissue damage and immunopathology in HMPV infection. Thus, future studies aimed at exploring pharmacologic modulators of TLR4–SLAMF1–p38 MAPK signaling may provide new opportunities to mitigate excessive TNF production and improve clinical outcomes in patients with severe HMPV infection.

## Data Availability

The raw data supporting the conclusions of this article will be made available by the authors, without undue reservation.
