# Supplementary material for: The immune receptors TLR4 and SLAMF1 regulate TNF release by human metapneumovirus in human macrophages
Source: Front Immunol. 2025 Nov 19;16:1697494. doi: 10.3389/fimmu.2025.1697494 (PMC12672276; doi:10.3389/fimmu.2025.1697494)
Supplement: Supplementary file 1 [file DataSheet1.docx]

Supplementary Material

# Supplementary Methods

## LDH assay for cell death evaluation

The percentage of cell death was assessed using the CyQUANT LDH Cytotoxicity Assay (#C20300, Thermo Fisher Scientific) to measure extracellular lactate dehydrogenase (LDH) released from damaged cells into the culture supernatant. THP-1 cells were seeded and differentiated as described in the *Materials and Methods* section and treated with BIRB796 or DMSO under the same conditions as used for HMPV infection (20 h treatment in Opti-MEM medium supplemented with 2% FBS). The assay was performed according to the manufacturer’s instructions, and results are presented as the percentage of dead cells (mean ± SD of biological replicates).

## Treatment with neutralising antibodies towards the IFN receptor (IFNAR)

The neutralising antibody anti-Human IFN-Alpha/Beta Receptor Chain 2, Clone MMHAR-2 (nIFNAR) was purchased from PBL Assay Science (#21385). Primary MDMs were incubated with nIFNAR (10 µg/mL) 30 min before infection with HMPV.

## Antibodies and reagents

The following antibodies were used for WB in supplementary material: rabbit phospho-MK2/MAPKAPK-2 (Thr334) (#3041), rabbit phospho-SAPK/JNK (Thr183/Tyr185) (#9251), rabbit phospho-ATF2 (T71) (#27934) from Cell Signalling Technology (Bionordika, Norway).

# Supplementary Figures and Tables

## Supplementary Figures

**Supplementary Figure S1**. **HMPV-induced CXCL10 expression is abrogated by type I IFN receptor neutralizing antibodies.**

Human MDMs were pretreated with or without IFNAR (IFN-α/β receptor) neutralizing antibodies (nIFNAR) before infection with HMPV (MOI 1) for 18 h and determination of *CXCL10* mRNA expression by qRT-PCR analysis. Results were normalized to non-infected (n.i.) samples. Data are presented as mean relative fold change ± SD for two independent experiments.


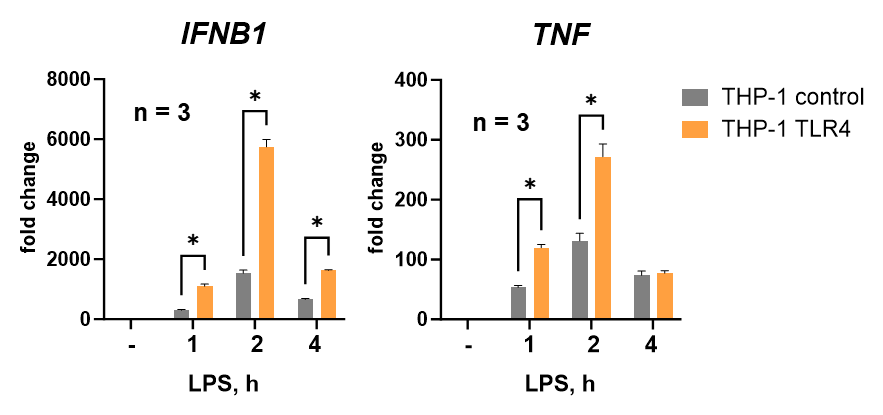


**Supplementary Figure S2. TLR4 overexpression results in the enhanced *IFNB1* and *TNF* mRNA expression in response to LPS stimulation.**

*IFNB1* and *TNF* mRNA expression was accessed by qRT-PCR in control and TLR4 THP-1 cells that were stimulated by LPS (100 ng/ml) for 2 h (n = 3). qRT-PCR results are normalized to non-stimulated (-) samples. Statistical testing was done by a multiple unpaired *t*-test with Welch correction (**p* < 0.05).

**
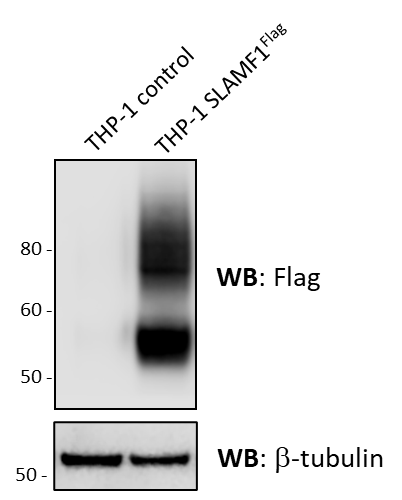
**

**Supplementary Figure S3. Flag-tagged SLAMF1 was overexpressed in THP-1 cells (THP-1 SLAMF1^Flag^) when compared to control cells that were transduced with empty pLVX vector (THP-1 control).**

Western blot analysis was performed using 10 µg of lysates of control and overexpressing cells, and primary antibodies towards the indicated proteins. WB for β-tubulin used as a loading control.


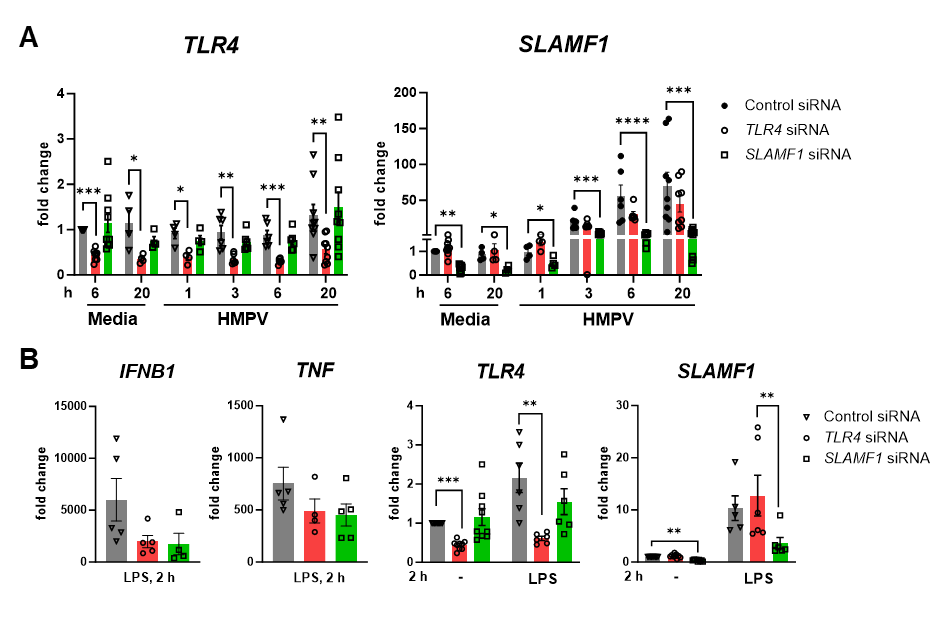


**Supplementary Figure S4. Silencing efficacy for *TLR4* and *SLAMF1* in primary MDMs was controlled by qRT-PCR, with LPS stimulation and qRT-PCR for cytokines used as functional positive control.**

(**A**) *TLR4* and *SLAMF1* mRNA expression for the samples shown on main Figure 5 was analyzed by qRT-PCR (n = 5-9). (**B**) Graphs show qRT-PCR analysis of *IFNβ* and *TNF* mRNA expression in MDMs stimulated by LPS (100 ng/ml) for 2 h, and *TLR4* and *SLAMF1* mRNA expression for the respective samples. (**A, B**). Results were normalized to non-infected and non-stimulated control sample (media 6 h). Data are presented as mean relative fold change ± SEM. Statistical testing was done by 2way ANOVA on log transformed data (**p* < 0.05, ***p* < 0.01, ****p* < 0.001, *****p* < 0.0001).

**
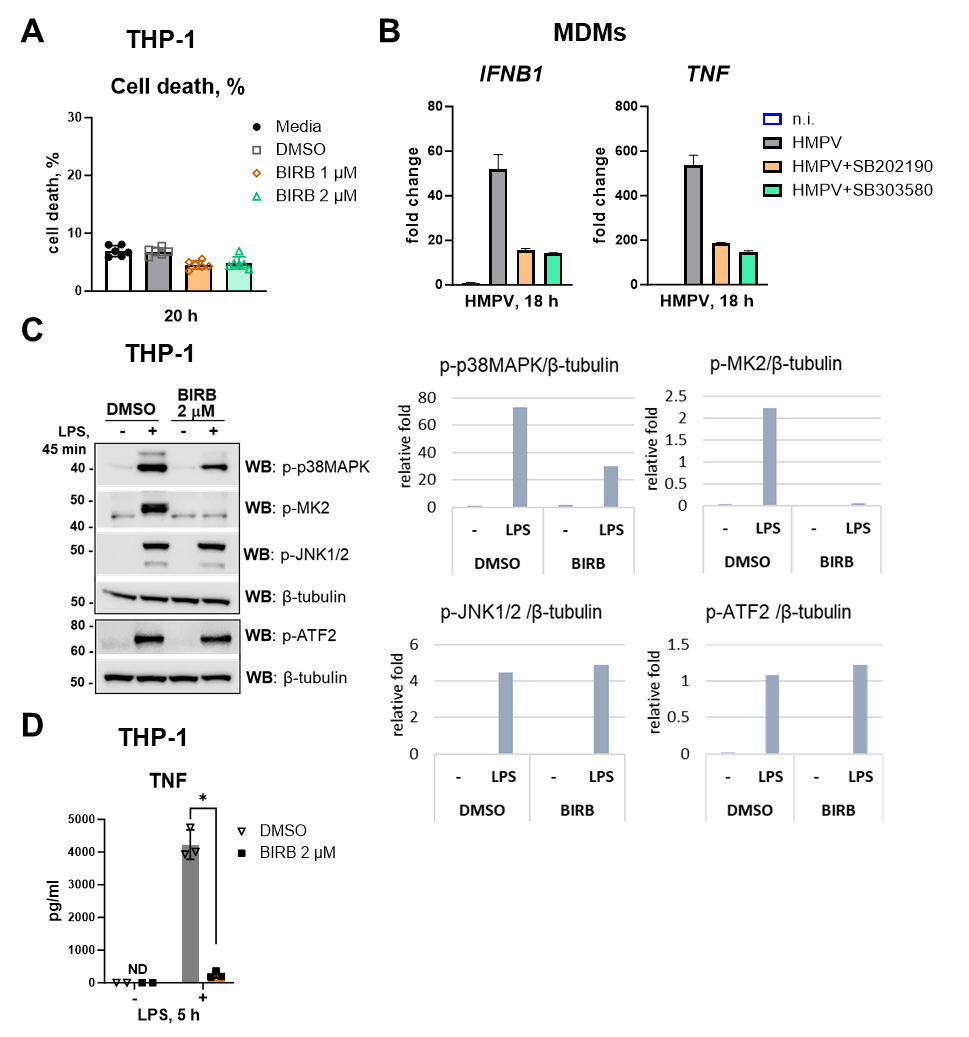
**

**Supplementary Figure S5. Cytotoxicity and specificity measures of BIRB796 and effect of the p38 MAPK inhibitors SB202190 and SB303580 on HMPV-induced *IFNB1* and *TNF* expression.**

(**A**) Cytotoxicity determination of BIRB796. THP-1 cells were differentiated with PMA and subsequently treated with the BIRB 796 inhibitor (1 or 2 μM), the corresponding highest concentration of DMSO, or left untreated for 20 h. All treatments were performed in Opti-MEM supplemented with 2% FBS, the same medium used for HMPV infection experiments. Supernatants were collected and analyzed using an LDH release assay to assess cell death. Data is shown as the percentage of dead cells (mean ± SD of biological replicates). (**B**) Effect of p38 MAPK inhibitors SB202190, or SB303580. *IFNB1* and *TNF* mRNA expression was determined by qRT-PCR in primary human MDMs pre-treated with DMSO (control), or 10 μM SB202190, or SB303580 p38 MAPK inhibitors for 30 min prior to HMPV infection (MOI = 1) for 18 h. Results were normalized to non-infected (n.i.) samples. (**C, D**) Specificity of BIRB796. PMA-differentiated THP-1 cells were pretreated with DMSO or BIRB796 inhibitor for 30 min, followed by LPS (100 ng/ml) stimulation for 45 min for WB analysis (**C**) or 5 h for TNF ELISA (**D**). The phosphorylation level of p38 MAPK (T180/Y182), MK2/MAPKAPK2 (T334), JNK1/2 (T183/Y185), ATF2 (T71) were evaluated by specific Abs, and normalized to level of β-tubulin (normalization is shown on graphs). **(D)** TNF secretion levels were examined by ELISA in the supernatants from unstimulated or LPS treated cells, with data presented as a mean ± SD (n = 3), ND – not detected. Statistical significance was evaluated using paired *t*-test, and only significant results are indicated (**p* < 0.05).
